# Merging National Forest and National Forest Health Inventories to Obtain an Integrated Forest Resource Inventory – Experiences from Bavaria, Slovenia and Sweden

**DOI:** 10.1371/journal.pone.0100157

**Published:** 2014-06-18

**Authors:** Marko Kovač, Arthur Bauer, Göran Ståhl

**Affiliations:** 1 Department of Forest and Landscape Planning and Monitoring, Slovenian Forestry Institute, Večna pot 2, Ljubljana, Slovenia; 2 Department of Food, Agriculture and Forestry Cham – Division of Forestry, Bavarian State Ministry of Food, Agriculture and Forestry, Ölbergstraβe 3, Waldmünchen, Germany; 3 Department of Forest Resource Management, Swedish University of Agricultural Sciences (SLU), Skogsmarksgränd, Umeå, Sweden; DOE Pacific Northwest National Laboratory, United States of America

## Abstract

**Backgrounds, Material and Methods:**

To meet the demands of sustainable forest management and international commitments, European nations have designed a variety of forest-monitoring systems for specific needs. While the majority of countries are committed to independent, single-purpose inventorying, a minority of countries have merged their single-purpose forest inventory systems into integrated forest resource inventories. The statistical efficiencies of the Bavarian, Slovene and Swedish integrated forest resource inventory designs are investigated with the various statistical parameters of the variables of growing stock volume, shares of damaged trees, and deadwood volume. The parameters are derived by using the estimators for the given inventory designs. The required sample sizes are derived via the general formula for non-stratified independent samples and via statistical power analyses. The cost effectiveness of the designs is compared via two simple cost effectiveness ratios.

**Results:**

In terms of precision, the most illustrative parameters of the variables are relative standard errors; their values range between 1% and 3% if the variables’ variations are low (s%<80%) and are higher in the case of higher variations. A comparison of the actual and required sample sizes shows that the actual sample sizes were deliberately set high to provide precise estimates for the majority of variables and strata. In turn, the successive inventories are statistically efficient, because they allow detecting the mean changes of variables with powers higher than 90%; the highest precision is attained for the changes of growing stock volume and the lowest for the changes of the shares of damaged trees. Two indicators of cost effectiveness also show that the time input spent for measuring one variable decreases with the complexity of inventories.

**Conclusion:**

There is an increasing need for credible information on forest resources to be used for decision making and national and international policy making. Such information can be cost-efficiently provided through integrated forest resource inventories.

## Introduction

The principle of sustainability [1], suggesting the use of forest resources in such a way as to continuously supply industry and households with wood assortments and fuel wood, was the main driving force that compelled European countries to begin inventorying their forestlands [2], [3]. Although the principle was understood similarly by the countries, different forest management traditions, practices and information needs compelled forest sciences and practices to develop forest inventories in two different directions.

The bottom-up stand-wise inventorying concept, promoted in Central Europe [4]–[7] and in other continents [8], was based on the idea that the data collected in small-sized allotments (viz. forest stands, compartments and forest management units) could be compiled upwards to support the information needs at larger planning scales (viz. forest management units, forest regions, nations). However, despite this theoretical possibility, in most cases, limited human and financial resources preventing the collection of detailed data in all management units, different data-collection techniques (e.g. full enumeration, purposive and probabilistic sampling, visual estimation) and the differences between the reference data hindered the derivation of statistically credible estimates at planning scales larger than forest management units [9]. Moreover, for the same reasons, these inventories could not provide reliable estimates on trends in time and space.

In contrast, the top-down inventory concept, developed in Nordic countries [10], [11], aimed to provide the basic estimates on forest resources such as the area, growing stock volume, increment, and so forth for large forest complexes. Unlike the first group of inventory concepts, this concept fully met the requirements of probabilistic sampling and consequently provided estimates with known levels of precision [3]. However, because of low sampling intensities, the usability of these estimates remained limited to large areas. Despite this deficiency, the theory behind these inventories was essential to all modern statistically-based forest inventories, especially to national forest inventories (hereafter: NFI).

Despite the shortcomings and rather poor sets of variables that aimed to describe forest sites, stands and forest resources (e.g. growing stock volume, increment), both inventory systems managed to provide information for formulating national forest policies, for international reporting [12], [13] and for creating forest management plans [9].

An important event that had implications for the development of the presently well-known integrated national forest resource inventories (especially in terms of comparable designs and multitudes of ecological variables) was the endorsement of the Convention on Long Range Trans-boundary Air Pollution [14]. Because the care for forest health was an essential element, the signatory states were invited to participate in the International Co-operative Program - Forests (hereafter: ICP Forests) and to build a large-scale cross-boundary forest health monitoring system. The system was to be used for detecting changes in tree and forest conditions over time and space and for better understanding the cause-effect relationships between the forest conditions and the factors affecting forest stands [15]. Although ICP Forests managed to build a joint inventory network covering the forests of all signatory states and to define the common sets of target variables along with the measurement protocols [15], it has neither succeeded in establishing close collaboration with the NFIs, nor in including their data in its analyses. Furthermore, the Life+ project [16], which, after the expiration of the Forest Focus scheme [17], was a kind of substitute for forest health monitoring, and whose actions were focused on the creation of a common sample grid and to the testing of methods for adapting data from NFIs, also had only partial success. Its greatest achievement was perhaps a scientific meeting in Florence in March 2009 (http://www.aisf.it/futmon/20.htm) at which a number of scientists described the progress, obstacles and future work in the field of inventory integrations.

A second event that potentially could contribute to the unification of forest inventory systems was the COST E43 action “Harmonization of National Forest Inventories in Europe: Techniques for Common Reporting” [18], [19], which was launched in 2004. Its objectives were (i) to improve and harmonize the existing national forest resource inventories in Europe, (ii) to support national teams in designing inventories to meet national, European and global requirements in supplying up-to-date, harmonized and transparent forest resource information, and (iii) to promote the use of scientifically sound and validated methods. The action supported three modules: Definitions and Measurement Practices, Estimation Procedures for carbon pools and carbon pool changes, and Indicators and Estimation Procedures of forest biodiversity [20].

Despite the general needs for integrated multi-resource forest inventories, which would not only bring the integrated data needed for complex statistical evaluations (viz. forest growth and yield, health and vitality [15], sustainable forest management [21], the assessment of the conservation status of forest habitat types and habitats [22]), but would also bring financial savings, the processes have only occasionally dealt with the questions of inventory integrations and have not, despite the many advantages of integrated inventories, compelled European countries to abandon the still prevailing single-purpose forest inventorying. Nevertheless, in addition to Switzerland and Austria, which integrated their inventories decades ago, some countries, among them the German state of Bavaria, Slovenia and Sweden, have managed to merge their independent, single-purpose forest inventory systems into integrated forest resource inventories.

In this article, we aim to present the methodological and organizational approaches that were used in designing the integrated forest resource inventories. We further aim to present the statistical features and the cost effectiveness of the three inventories. Finally, we discuss the state-of-the-art in the field of integrated forest resource inventories in Europe and North America, from which the latest innovations come.

## Materials, Methods, Integration Patterns

### Ethics Statement

All the results come from public data. No ethic issues have been violated.

### Material, Working Methods

The presented results come from the NFI and the national forest health inventory datasets of Bavaria, Germany, Slovenia and Sweden [23]–[25]. The same datasets are used as the official data-sources for national and international reporting [26] and for other purposes.

The statistical efficiencies, related to the statistical precisions and to the abilities of the three designs to detect changes, were investigated by analyzing three variables (Table 1): growing stock volume (hereafter: GSV), share of damaged trees (trees defoliated more than 25%; hereafter: ShDT), and deadwood volume (hereafter: DWV). These variables were chosen because i) they have been monitored by all the three inventory systems, ii) they have been recognized as the core indicators by several international processes [21], [15], [27], and iii) they generally have rather different variability, which definitely affects the efficiencies of integrated forest resource inventories. Apart from the share of damaged trees that has been defined by ICP Forests [28], the other two variables have remained non-harmonized.

**Table 1:** Core variables and their definitions as used in the NFIs of Bavaria, Germany, Slovenia and Sweden.

Country	Variable	Definition	Thresholds	Method/Reference	Inventory year/cycle	Remarks
Bavaria/Germany	GSV (m^3^/ha)	Standing alive trees,volume over bark,stump included	DBH≥10 cm	[23]	1987	
			DBH≥7 cm	[41]	2002, 2012	
Slovenia	GSV (m^3^/ha)	Standing alive trees,volume over bark,stump included	DBH≥10 cm	[42]	1985, 1991,1995, 2000	
			DBH>0 cm		2007	
Sweden	GSV (m^3^/ha)	Standing alive trees,volume over bark,stump excluded	DBH>0 cm	[10]	Continuousinventory	Estimates calculatedon 5-year averages.
Bavaria/Germany	ShDT(%)	% of damaged trees	Trees with anassessed defoliationof 25% or more	[28]		
Slovenia	ShDT(%)	% of damaged trees	Trees with anassessed defoliationof 25% or more	[28]	1985, 1991,1995, 2000,2007	Continuous inventoryon the 16×16 km grid
Sweden	ShDT(%)	% of damaged trees	Trees with anassessed defoliationof 25% or more	[28]	Continuousinventory	Estimates calculatedon 5-year averages.
Bavaria/Germany	DWV (m^3^/ha)	Dead standing anddowned trees,snags, stumps,coarse woody debris	Standing deadwood:DBH≥20 cm	[41]	2002	
			Stumps: D≥50 cmor H≥50 cm			
			Snags: D≥20 cm;L without threshold			
Slovenia	DWV (m^3^/ha)	Dead standing anddowned trees,snags, stumps,coarse woody debris	Standing deadwood:DBH≥10 cm	[42]	2007, 2011	
			Stumps: D≥10 cmand H≥20 cm			
			Snags and coarsewoody debris:D≥10 cm,L≥50 cm			
Sweden	DWV (m^3^/ha)	Dead standing andowned trees, snagsand coarse woodydebris; stumps excluded	Standing deadwood:DBH>4 cm	[10]	Continuousinventory	Estimates calculatedon 5-year averages.
			Lying dead wood:DBH>10 cmand 1.3 m long			

GSV = growing stock volume; ShDT = share of damaged trees; DWV = deadwood volume; DBH = diameter at breast height.

The statistical parameters of the three variables (i.e. means, variances and confidence intervals) were derived by using the estimators for the given inventory designs [2], [10], [29], [30], [42], [66]. The required sample sizes were derived by:

- the confidence interval, also known as the general formula for non-stratified independent samples [2], [30] n = (t*s%/E%)^2^
  whereby t = t statistics, s% = coefficient of variation, E% = relative standard error (also relative precision or relative allowable error), and also through
- statistical power analyses [31]. The latter approach was used to further explore the sample sizes of the variables at given different values of statistical power. The power analysis was employed in three ways:
  - the choice “independent sample t-test H0: M1 = M2” (whereby H0 = null hypothesis; M1 = sample mean at the first occasion; M2 = sample mean at the second occasion) was used for calculating the sample sizes of the independent variables (e.g. cut time series);
  - the choice “dependent sample t-test H0: M1 = M2” (whereby H0 = null hypothesis; M1 = sample mean at the first occasion; M2 = sample mean at the second occasion) was used for calculating the sample sizes of the paired variables (viz. GSV and ShDT as continuous time series); and
  - the choice “one mean t-test H0: M1 = M0” (whereby H0 = null hypothesis; M1 = sample mean; M0 = hypothesized mean) was used for calculating the sample sizes of the variables that had been measured or assessed only once (e.g. the first measurement of deadwood).

In judging the overall statistical efficiencies of the designs, a single variable approach was used [29].

The cost effectiveness of the designs was compared via simple cost effectiveness ratios, such as CER (cost effectiveness ratio) and ICER (incremental cost effectiveness ratio) [32], [33] as follows:
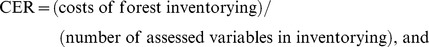





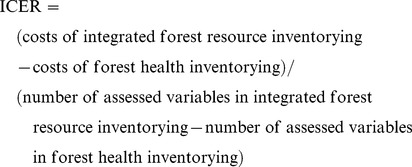



where by as the costs of inventorying were used the average total time inputs taken from the field-forms of arbitrary inventorying (viz. forest health, integrated forest resource inventorying) and, as the number of assessed variables, the numbers of variables assessed by each type of inventorying were used. The average total time input includes: transportation from the office to a cluster, walk from the car to the cluster and back, walk between the plots in the cluster, field measurements, transportation from one cluster to another and transportation back to the office.

The CER index was used for ranking or comparing the cost-effectiveness of mutually independent inventories, and the ICER index was used for a comparison of the cost effectiveness of mutually exclusive forest inventories. The latter approach can also be understood as the approach of marginal costs.

All computation was carried out by using the Statsoft (v.10) statistical modules [34].

The presentation of the historical developments of the inventory designs required no specific methodology. As the basic sources, we used the relevant published literature.

### Historical Development of the Inventory Designs

#### Bavaria (Germany)

**Figure 1:**
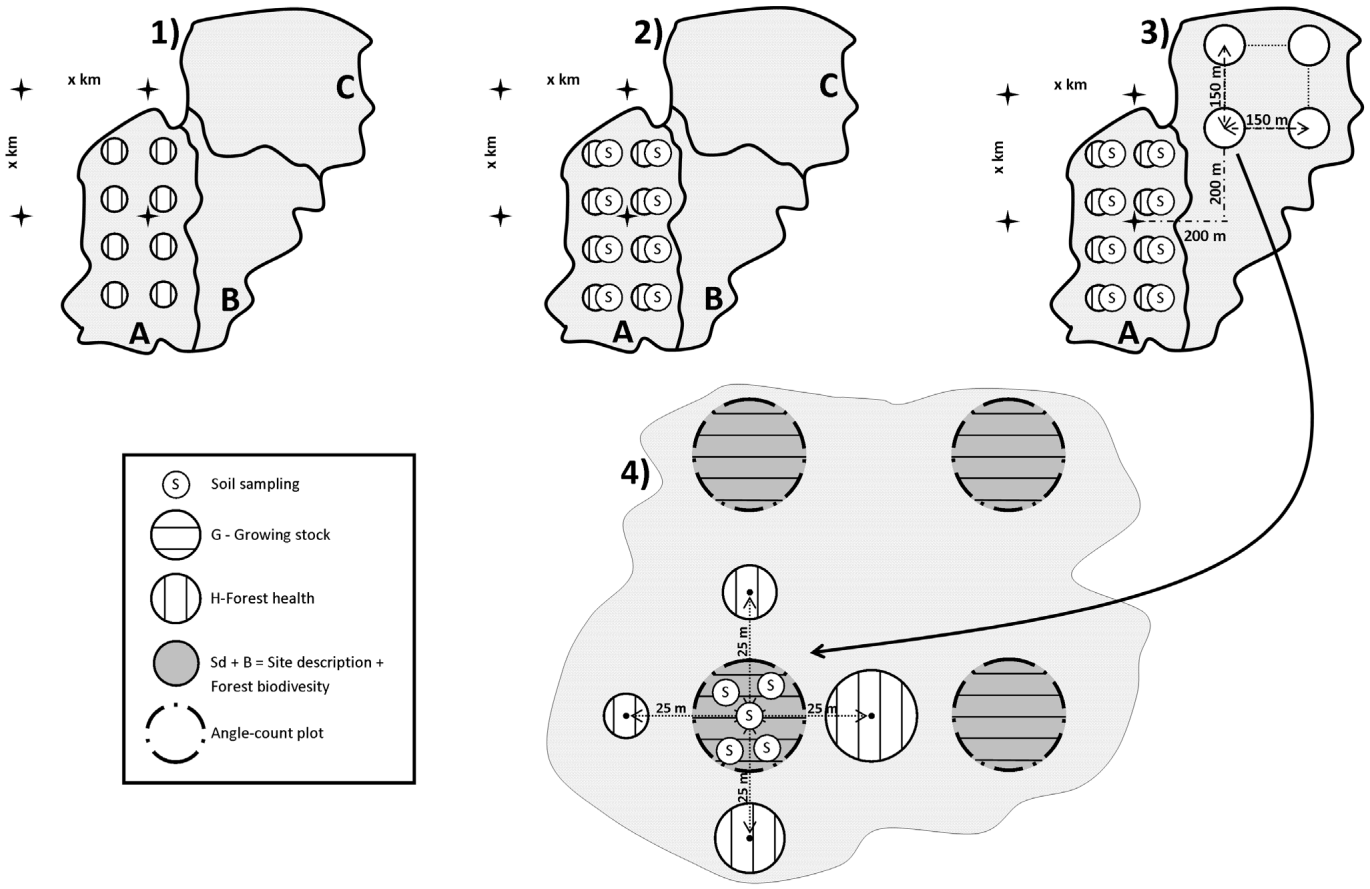
Historical development of Bavaria’s integrated forest resource inventory. 1) 1983–1985: National Forest Health inventory. The inventorying was performed in angle-count plots, evenly distributed across forest stands. 2) 1986: National Forest Health and Forest Soil Inventory. Soil plots, used for soil sampling, were distributed in parallel to the forest health plots. 3) 1986–2005. National Forest Inventory. The introduction of the NFI clusters, consisting of four angle-count plots, distributed across forest complexes. 4) 2006–2008. Integrated Forest Resource Inventory (IFRI). Forest soil sampling and forest health assessment, performed on the subsamples of the NFI/IFRI plots (southwestern plot on the 8×8 km grid), have become part of the inventory design. The majority of variables has been collected on the angle-count plots (NFI/IFRI plots), while forest health has been assessed on the 6-tree plots.

Bavaria began statistically-based large-scale forest inventorying in 1983 (Table 2), when it launched (along with the neighboring state of Baden-Wuerttemberg) its forest health inventory. In terms of statistical design, it was a systematic cluster-sampling inventory, whereby the systematically arranged grid of 4×4 km defined forest stands, which were sampled with clusters of six to ten (Figure 1) systematically arranged angle-count plots [35]. In concert with the ICP Forests Manual [28], only the trees belonging to the Kraft classes 1, 2 and 3 were selected. The computations were based on the time series of the tree data, meaning that harvested and naturally died trees, as well as the trees unsuitable (e.g. broken, bent) for assessments, were continuously replaced with the trees closest to the center of a plot [35].

**Table 2:** Historical development of large-scale forest resource inventory in Bavaria, Germany.

Year	Inventory type	Grid density (km)	Number of clusters/plots (Type of plots)	Major variables	Major improvements/Comments
1983–2005	NFHI	4×4	Approx. 1700 clusters(angle- count plots)	Characteristics of foreststands; defoliation; damages;mortality;	6 to 8 plots within forest stands;Trees chosen by angle-count-sampling [35]. No data assessmentin 1990 due to heavy damages causedby the storms Vivian and Wiebke.
	FHI_ICP	16×16	123 plots(angle-count plots)		
1986–1988	NFI I	4×4; in some areas2.83×2.83 and 2×2	3279 clusters (cluster150×150 m with fourangle-count plots)	Growing stock, regeneration,forest openness, sitecharacteristics (site form,site gradient, site aspect);	Introduction of NFI grid (4×4 km)and NFI clusters in parallel(200 m westward and northward)to the old NFHI grid [43].
2001–2002	NFI II	4×4; in someareas 2.83×2.83	Approx. 2700 clusters(cluster 150×150 mwith four angle-count plots)	Growing stock, regeneration,site characteristics (site form,site gradient, site aspect),deadwood, naturalness;	Cluster with the side lengths of150 m; Sample trees are chosenby angle-count sampling at eachplot of the cluster.
1987	NSI I	8×8	424 clusters (ten sub-plotsdistributed over theforest stand)	Soil types, acidity, carbon,soil water, humus, nitrate,base saturation, heavy metal;	Sample plots are located next tothe plots of ICP-plots; the centerof the plots is unmarked [36].
2006–2008	NSI II	8×8	386 clusters (five samplesquares/cluster)	Soil types, acidity, carbon,soil humidity, humus, nitrate,base saturation, heavy metals;	
2006	IFRI III	8×8	386 clusters (four angle-count and four 6-tree plots)	Growing stock, regeneration,site characteristics (site form,site gradient, site aspect),deadwood, naturalness; soiltypes, acidity, carbon, soilhumidity, humus, nitrate,base saturation, heavy metals;	The ICP and the soil sample plotsare installed on the southwestcorner of the NFI-cluster;The ICP plots are installedaccording to the ICP-ForestsManual [28].

FHI_ICP = Forest health inventory for the needs of the ICP (only variables related to forest health); NFHI = National Forest Health Inventory (only variables related to forest health); NSI = National Forest Soil Inventory (soil variables); NFI = National Forest Inventory (conventional forest management variables - without forest health variables); IFRI = Integrated forest resource inventory (all variables viz. forest health, forest area, forest growth and yield, biodiversity, carbon-sequestration, forest soil, etc.); CPSP = circular permanent sample plot.

The same inventory design was also used at the occasion of First Bavaria’s Forest Soil Inventory in 1986, whose aim was to detect the radioactive fallout caused by the Chernobyl nuclear power plant disaster as well as to determine the rates of forest sites’ nutrition and contamination [36]. As shown in the figure, the soil plots were located in parallel to the forest health plots.

A shift from the described development of the national forest monitoring system occurred in 1986 when the nation, including Bavaria, underwent the first statistically-based national forest inventorying [66]. Despite the existing forest health permanent sample plot (hereafter PSP) system, Bavaria did not use it, but rather installed a parallel system of clusters, each consisting of four angle-count plots. The main reason that a new system of clusters was introduced (200 m eastward and northward of the existing forest health grid, Figure 1) was the possibility of biased results due to the visibly marked sample trees used in forest health monitoring. This forest inventorying was conducted until 2005.

Another change of the inventorying system occurred in 2006 when the German states set up the Second National Forest Soil Inventory. Although the inventory should have been a repetition of the first inventory of 1986, Bavaria moved the clusters of PSP for soil sampling to the NFI grid. The main reason was the fact that the soil sampling plots were not marked at the occasion of the first inventory. Finally, the last improvement came with the latest design for forest health inventory which followed the ICP-Forests proposal [28]. The clusters were composed of four 6-tree plots [37] and were installed on the subset of the NFI grid. All the previously used plots were abandoned.

#### Slovenia

**Figure 2:**
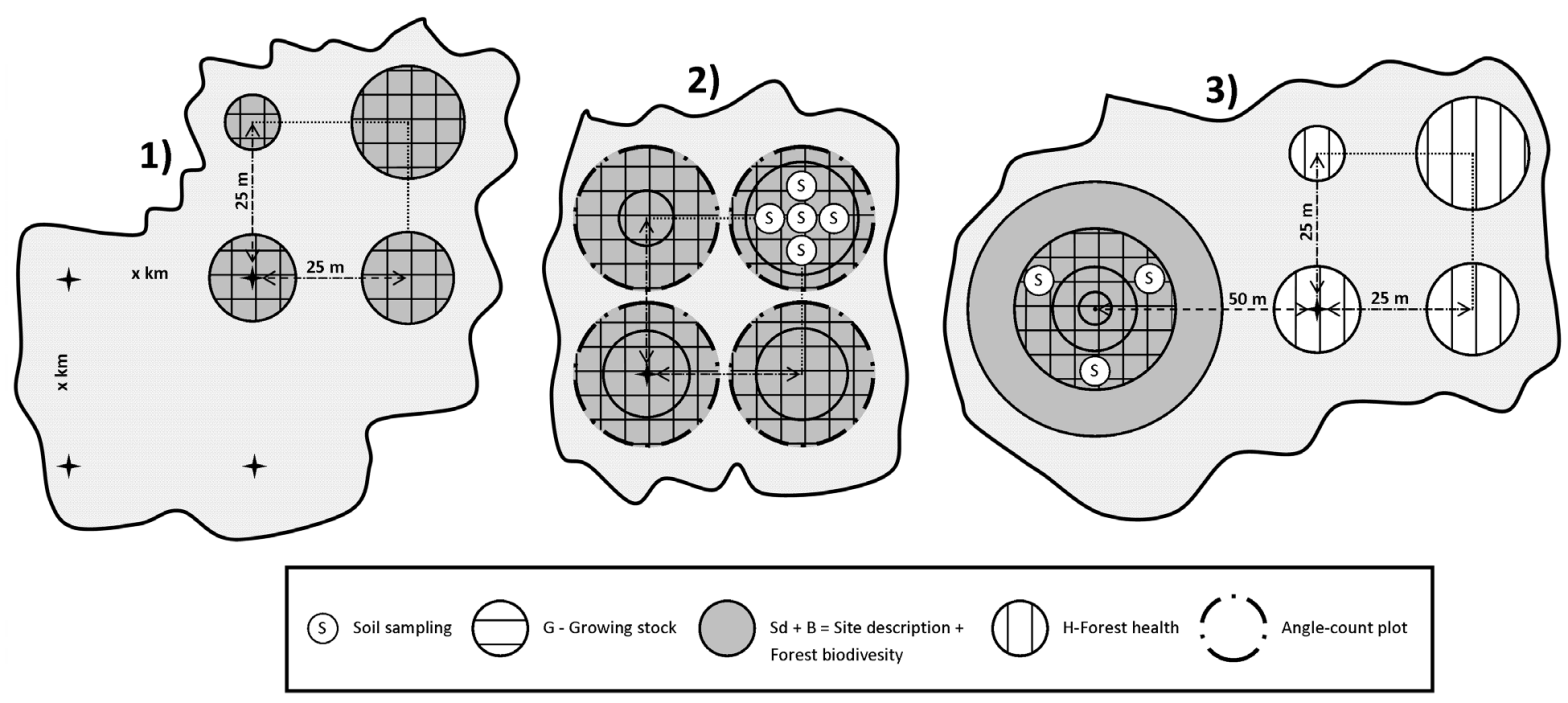
Historical development of Slovenia’s integrated forest resource inventory. 1) 1987–1994: National Forest Health Inventory. The inventorying was performed in clusters, consisting of four 6-tree plots. 2) 1995: Integrated Forest Resource Inventory (IFRI). Forest soil sampling and the assessment of growing stock became part of inventorying. To achieve good estimates on growing stock, angle-count plots (superimposed over 6-tree plots) were introduced. 3) 2000 onwards: Integrated Forest Resource Inventory. Introduction of invisible concentric permanent sample plots with fixed areas. The plots have been used for collecting data and information on forest sites, growing stock and increment, forest health, biodiversity, ecosystem services, average tree cut (harvest). Forest health has been assessed on 6-tree plots and on concentric permanent sample plots.

Until the 1980s, Slovenia was obtaining forest resources information at a national level through the bottom-up inventorying concept [9]. A statistically sound large-scale forest health inventory design (cluster sampling with clusters consisting of four 6-tree plots [37]) was first introduced in 1985 (Table 3), when the country entered the UN/ECE ICP Forests program [38]. In addition to the required tree health data [28], the surveying also provided data on forest vegetation types, site and stand conditions, tree-species composition and so forth. In 1995, this inventory design was improved with angle-count plots [37] that were superimposed over the 6-tree plots (Figure 2). Both estimates (derived by angle-count sampling and by 6-tree sampling method) enabled the derivation of an objective estimate of the growing stock volume for the first time [39].

**Table 3:** Historical development of large-scale forest resource inventory in Slovenia.

Year	Inventory type	Grid density (km)	Number of clusters/plots(Type of plots)	Major variables	Major improvements/Comments
Since 1985	FHI_ICP	16×16	40–43 clusters(four 6-tree plots/cluster)	Forest site, tree-species,defoliation, mortality, damages;	
1985and 1991	NFHI	4×4	700–800 clusters(four 6-tree plots/cluster)	Forest site, stand features,tree-species, defoliation, mortality,damages, DBH;	Growing stock volume andincrement volume werenot computed.
1995	NFI I	4×4	712 clusters (four 6-tree plots/cluster+four angle-count plotssuperimposed over 6-tree plots)	Forest site, stand features,tree-species, defoliation, mortality,damages, DBH, height, lichens;	Update of the number of clustersIntroduction of angle-count plots(overlaid over 6-tree plots);Calculation of growing stockand increment volume.
2000	IFRI I	4×4	712 clusters (one CPSPand two 6-tree plots/cluster)	Forest site, stand features,tree-species, defoliation, mortality,damages, DBH, height,age-estimate, lichens,deadwood (without stumps);	Update of the number of clusters;Introduction of CPSP; Changeof the clusters’ standpoints.
2007	IFRI II	4×4	737 clusters; (one CPSPand two 6-tree plots/cluster)	Forest site, stand features, tree-species, defoliation, mortality,damages, DBH, height, age-estimate, lichens,deadwood(with stumps), small trees;	Calculation of increment volume;Introduction of soil variables.
		Soil sampling: 8×8	Sample squares	Organic horizon, mineral partof soil, pH, C/N, bulk density;	
2012	IFRI III	4×4 Non-forestland:sample from the1×1 km grid	a) 762 clusters+509 non-forest clusters(one CPSP/cluster)	Forest site, stand features,tree-species, mortality, DBH,height, age-estimate, deadwood(with stumps), small trees;Organic horizon, mineral partof soil, pH, C/N, bulk density;Non-forest area:Wooded area, DBH, height,organic horizon, mineral partof soil, pH, C/N, bulk density;	Updated clusters; Extension ofinventorying to non-forest area(other wooded land, orchards,grasslands).

FHI_ICP = Forest health inventory for the needs of the ICP (only variables related to forest health); NFHI = National Forest Health Inventory (only variables related to forest health); NSI = National Forest Soil Inventory (soil variables); NFI = National Forest Inventory (conventional forest management variables - without forest health variables); IFRI = Integrated forest resource inventory (all variables viz. forest health, forest area, forest growth and yield, biodiversity, carbon-sequestration, forest soil, etc.); CPSP = circular permanent sample plot.

However, because 6-tree plots had been rather inconvenient for the long-term tracking of changes (due to the variability of plot sizes, different inclusion probabilities of trees and the exchange of trees on the plots [65]) and would have shortly become unrepresentative due to visibly marked trees, the design was changed in the year 2000 [40], by adding invisibly-marked circular permanent sample plots (hereafter: CPSP) to the existing clusters. The main features of this design are: i) compliance with the principles of random sampling; ii) open clusters [30] requiring one day’s work of a crew; iii) possibility of tracking the historical development of the CPSPs; iv) possibility of using various statistical techniques; v) ability to use the time series from the past, and; vi) openness to future modifications.

Despite improvements, the 2007 inventory has not fully covered the needs of end-users. Consequently, the surveying carried out in 2012 took account of non-forestland to provide estimates on the biomass outside forestland. Many improvements are yet to come; hence, future modifications will have to address the optimization of the sample grid to provide a variety of estimates of different variables for the strata and regions [44], as well as the temporary sample plots to control the representativeness of the installed CPSPs, indicators of ecosystem services and so forth.

#### Sweden

**Figure 3:**
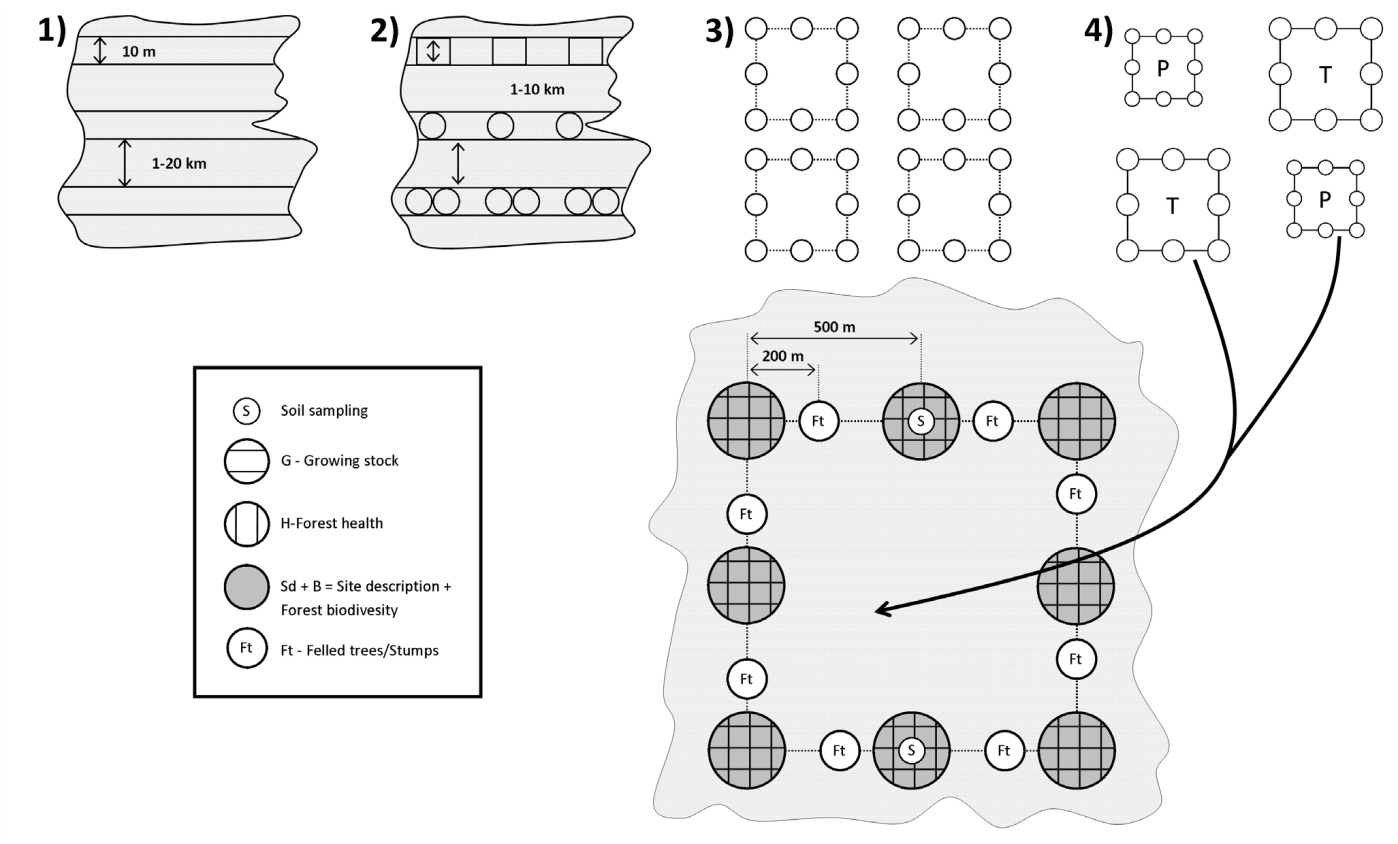
Historical development of Sweden’s integrated forest resource inventory. 1) 1923–1929: County-by-county forest inventory. Full callipering in strips. 2) 1938–1952: County-by-county forest inventory. Introduction of squared and circular sample plots. 3) 1953–1982: NFI. Introduction of sample clusters (tracts). 4) 1983 onwards: Integrated Forest Resource Inventory (IFRI). Introduction of permanent (P) and temporary (T) clusters (tracts) of different sizes, used for inventorying forest areas, growth, biodiversity, soil condition, forest health. 5) 1983–2006. National Forest Health Inventory. Forest health assessment was performed on one NFI/IFRI plot of selected permanent tracts. 6) 2007 onwards: Forest health assessment has been performed on all NFI/IFRI plots. Trees have been selected via angle-count method.

NFIs have a long tradition in Sweden (Table 4). They emerged in the 1920s as regional inventories, proceeding county by county until the entire country was covered [45]. Later, the importance of timely information about the state of and changes in forestry conditions for the entire country was recognized, and the NFIs were rearranged into a system in which a sample of the entire country was selected each year. Statistical developments of this process were documented by Matérn [46]. From the 1950s onwards, the basic set-up of Swedish NFIs has remained fairly constant (Figure 3), although slight modifications are made every five to ten years. The main features of the Swedish system are: i) compliance with the principles of random sampling; ii) employment of clusters of plots, corresponding to one day’s work for a field crew; iii) adaptation to varying conditions in different parts of the country, partly through a stratified design in which details of the design are regionally optimized; iv) a combination of permanent (visited every five years) and temporary plots; v) openness to modifications of the plot-level measurement protocol.

**Table 4:** Historical development of large-scale forest resource inventory in Sweden.

Year	Inventory type	Grid density (km)	Number of clusters/plots(Type of plots)	Major variables	Major improvements/Comments
1923–1929	NFI I	Strip inventory	Different in different regions	Growing stock, stand andsite characteristics;	
1935–1952	NFI II	Mix of stripsand plots	Different in different regions	Growing stock, stand andsite characteristics;	Introduction of sample plotsinstead of sample strips.
1953–1982	NFI III-V	Unique for each of5 different strata	Unique for each of 5different strata	Growing stock, stand andsite characteristics;	Introduction of sample plotclusters, so-called tracts.
1983–1992	NFI VI	Unique for each of5 different strata	Unique for each of 5different strata	Growing stock, stand and sitecharacteristics, as well as soilclassification and detailed vegetationvariables;	Introduction of permanentplots and a specific soil survey.
1993–2002	NFI VII	Unique for each of5 different strata	Unique for each of 5different strata	Growing stock, stand and sitecharacteristics, as well as soilclassification, detailed vegetationvariables, crown condition, andbiodiversity indicators;	Introduction of several newvariables to capture biodiversityand forest damage.
1994–2006	FHI_ICP	16×16 km	770 plots	Crown condition and forestdamage; annual assessments;	ICP Forests plots selected asa subset of permanentplots of the NFI.
2003–2013	IFRI VIII	Unique for each of5 different strata	About 10000 plots measuredannually (allocation differentin each of 5 different strata)	Growing stock, stand and sitecharacteristics, as well as soilclassification detailed vegetationvariables, crown condition,forest damage, and biodiversityindicators;	ICP Forests Level I and NFImerged From 2007 onwards.

FHI_ICP = Forest health inventory for the needs of the ICP (only variables related to forest health); NFHI = National Forest Health Inventory (only variables related to forest health); NSI = National Forest Soil Inventory (soil variables); NFI = National Forest Inventory (conventional forest management variables - without forest health variables); IFRI = Integrated forest resource inventory (all variables viz. forest health, forest area, forest growth and yield, biodiversity, carbon-sequestration, forest soil, etc.); CPSP = circular permanent sample plot.

In the 1990s, the ICP Forests Level I plots were installed as a subset of the NFI permanent plots. In this way, 770 plots were randomly distributed throughout the country; plots were selected (one plot per selected cluster) so that a fairly systematic grid of Level I plots was obtained across the country. Until 2006, the Level I plots were visited each year, and registrations of crown condition and tree damage parameters were made. Basic data about site conditions, growing stock, etc., were taken from the NFI measurements.

From 2007 onwards, the annual measurements of the Level I plots were abandoned and crown condition data were instead only taken from the ordinary NFI plots. On all the NFI plots, certain (sub-) sample trees are selected for more careful measurements. These trees are selected through probability to size (basal area) sampling, and normally about two to five trees per plot are selected. For these trees, in addition to traditional measurements, such as height, crown height, stem quality, etc., assessments of crown condition variables are made. On the basis of these measurements, statistical estimates of crown condition parameters are computed.

Although the basic cluster design remains the same throughout the country, several regional differences exist. In southern Sweden, dense grids with small clusters are used, whereas in northern Sweden sparse grids with larger (more plots) clusters are applied. The smallest clusters in southern Sweden comprise four plots, whereas the largest clusters in northern Sweden comprise sixteen plots. The clusters generally are squares with the side lengths ranging from about 200 meters to 1.600 meters. The plots are concentric, with most of the tree-related measurements conducted within a 10-meter (permanent plots) or 7-meter (temporary plots) radius. Slightly more than 10.000 plots are sampled every year; about two thirds of these are permanent.

## Results

Tables 5 and 6 provide an overview of the statistical efficiencies of the three inventory designs. Among the many parameters of the variables presented in Table 5, the relative standard errors are perhaps the most illustrative; their values range between 1% and 3% if the variations of the variables are low (s%<80%) and are much higher if the variables have larger variations. Furthermore, a comparison of the actual (n_plots_) and the required sample sizes (n_gf_) leads to the conclusion that the actual sample sizes (especially of Bavaria and Sweden) are being set deliberately high to provide precise estimates for all variables (not only for target ones), and for diverse strata.

**Table 5:** Basic statistics of core variables.

Old inventory system									Current inventory system								Remarks
C/R	Var	Y	n	M	s	s%	E	E%	n	M	s	s%	E	E%	E%_all_	n_gf_	
BavG	GSV (m^3^/ha)	1987							3279	333.00	267.00	80.20	4.66	1.40	5	988	(including bark)
BavG	GSV (m^3^/ha)	2002							2622	403.00	206.00	51.10	4.02	1.00	5	401	(including bark)
SLO	GSV (m^3^/ha)	1995	712	283.17	152.69	53.92	5.72	2.02									[39]
SLO	GSV (m^3^/ha)	2000							712	281.39	173.48	61.65	6.50	2.31	5	584	[47]
SLO	GSV (m^3^/ha)	2007							737	323.60	193.98	59.94	7.14	2.21	5	552	[47]
SWE	GSV (m^3^/ha)	2000							8450	108.80	90.01	82.73	0.98	0.90	5	1052	
SWE	GSV (m^3^/ha)	2007							6930	114.30	85.64	74.93	1.03	0.90	5	863	
BavG	ShDT (%)	2005	202	22.70	14.20	56.40	1.00	4.40									
BavG	ShDT (%)	2006							386	22.70	12.90	55.80	0.66	2.89	5	478	
SLO	ShDT (%)	1995	680	24.69	18.37	74.40	0.70	2.84									
SLO	ShDT(%)	2000	677	24.72	20.56	83.17	0.79	3.20	683	22.19	17.52	78.96	0.67	3.02	5	958	
SLO	ShDT(%)	2007							726	39.63	25.00	63.09	0.93	2.35	5	611	
SWE	ShDT(%)	2000	770	29.30	30.00	102.39	1.08	3.69									Spruce
SWE	ShDT(%)	2007							1386	34.30	30.00	87.46	0.79	2.30	5	1175	Spruce
SWE	ShDT(%)	2000	770	14.30	25.00	174.83	0.90	6.29									Pine
SWE	ShDT(%)	2007							1386	15.00	25.00	166.66	0.67	4.48	5	4268	Pine
BavG	DWV (m^3^/ha)	2002							1280	12.90	5.60	43.40	0.16	1.24	5	289	
SLO	DWV (m^3^/ha)	2007							737	18.64	36.36	195.05	1.34	7.19	10	1461	
SLO	DWV (m^3^/ha)	2007													15	650	
SWE	DWV (m^3^/ha	2000							8450	6.50	11.95	183.85	0.13	2.00	5	5193	
SWE	DWV (m^3^/ha	2007							6930	8.30	13.82	166.51	0.17	2.00	5	4260	

C/R = country/region; BavG = Bavaria_Germany; SLO = Slovenia; SWE = Sweden; Var = Variable; Y = year; n = number of plots; M = mean value; s = sample standard deviation; s% = coefficient of variation; E = standard error; E% = relative standard error; E%_all_ = allowable relative standard error; n_gf_ = n computed by general formula for non-stratified sampling n = (1.96×s%/E%_all_)^2^; See also abbreviations in Table 1.

**Table 6:** Statistical efficiencies of the designs.

InvC	Var	Y	n	M	P_c_ (%)	Required n at given power (P_set_) and % of mean change							InvPair	Test type
						P_set_	±2%	±5%	±10%	±15%	±20%	±25%		
BavG_1	GSV	1987	3279	333.00										
BavG_2a	GSV	2002	2622	403.00	100	0.99		3842	962*	428*			BavG_1-2a	2M IDP
BavG_2b	GSV	2002	2622	403.00	100	0.90		2198*	551*	246*			BavG_1-2b	2M IDP
SLO_1	GSV	2000	712	281.40										
SLO_2	GSV	2007	737	323.60	100	0.99		529*	134*	61*			SLO_1-2	2M DEP
SWE_1	GSV	2000	8450	108.80										
SWE_2a	GSV	2006	6930	114.30	100	0.99	7879	1285*	323*				SWE_1-2a	2M DEP
SWE_2b	GSV	2006	6930	114.30	100	0.90	4507*	736*	186*				SWE_1-2b	2M DEP
BavG_1a	ShDT	2006	386	22.70	93	0.99		2529	580	167*			BavG_1a	1M
BavG_1b	ShDT	2006	386	22.70	93	0.90		1447	333*	154*			BavG_1b	1M
SLO_1	ShDT	2000	683	22.20										
SLO_2a	ShDT	2007	726	39.60	100	0.99		3620	907	418*			SLO_1-2a	2M DEP
SLO_2b	ShDT	2007	726	39.60	100	0.90		2071	520*	240*			SLO_1-2b	2M DEP
SWE_1	ShDT	2000	770	29.30										
SWE_2a	ShDT	2007	1386	34.30	96	0.99		5724	1433	638*			SWE_1-2a	2M DEP
SWE_2b	ShDT	2007	1386	34.30	99	0.90		3275	820*	366*			SWE_1-2b	2M DEP
SWE_1	ShDT	2000	770	14.30										
SWE_2a	ShDT	2007	1386	15.00		0.99			5106	2271	1278*		SWE_1-2a	2M DEP
SWE_2b	ShDT	2007	1386	15.00		0.90			2921	1300*	732*		SWE_1-2b	2M DEP
BavG_1a	DWV	2002	1280	12.90	98	0.99		1603	343*	162*	153*		BavG_1a	1M
BavG_1b	DWV	2002	1280	12.90	98	0.90		918*	197*	94*	89*		BavG_1b	1M
SLO_1a	DWV	2007	737	18.60	93	0.99				3107	1781	1126	SLO_1a	1M
SLO_1b	DWV	2007	737	18.60	93	0.90			3859	1778	1017	645*	SLO_1b	1M
SWE_1	DWV	2000	8450	6.50										
SWE_2a	DWV	2007	6930	8.30	100	0.99		10062	2517*	1120*			SWE_1-2a	2M DEP
SWE_2b	DWV	2007	6930	8.30	100	0.90		5755*	1441*	642*			SWE_1-2b	2M DEP

InvC = inventory code; Var = Variable; Y = Year; n = number of plots; M = mean; P_c_(%) = computed power for independent and dependent samples and for the hypothesized mean; P_set_ = set power; Required n at given power (P_set_) and % of mean change = required sample size at 2%, 5%, 10%, 15% …change in mean; InvPair = inventory pair; 2M IDP = two mean test for independent samples; 2M DEP = two mean test for dependent samples; 1M = one mean test (of hypothesized mean);

* = actual sample size successfully detects the differences in mean change. See also abbreviations in Table 1.

A more detailed overview of the statistical efficiencies is provided in Table 6, which presents the results of the power analysis. The first finding is that the successive inventories have been statistically very efficient, because the actual mean changes of the given variables have been detected with statistical powers higher than 90% (column P_c_). Also informative are the simulations of mean changes of the three variables (set to 5%, 10% change etc.), which, at the set powers of 90% and 99% respectively, reveal that the existing designs aim to be multi-resourceful as they enable detecting the mean changes of the variables with a highly diverse variation; in the case of GSV, all the three designs allow the detection of 5% and larger mean changes at the power of 90% and the mean changes of 10% and larger at the power of 99%. The Swedish inventory is even more efficient, as it allows detecting a 2% mean change at the power of 90%. Less precise is the detection of mean changes of the variable ShDT; the power of 90% mostly allows the detection of mean changes to the extent of 10% and more, while the power of 99% enables detecting the mean changes to the extent of 15% and more. Similar effectiveness of the designs is also experienced at detecting changes in DWV. However, the exploration of changes of this variable suggests improvements. This is especially so for the Slovenian inventory, which needs a denser grid to become more effective in change detection.

Table 7 presents two cost effectiveness indicators for two different types of inventories. The first indicator, CER, clearly shows that the time input spent for measuring one variable decreases with the complexity of inventories; the more variables to be recorded, the smaller time input per variable needed. Additionally, the second indicator, ICER, shows that by implementing integrated (merged) multi-purpose instead of single-purpose inventories (e.g. forest health versus integrated forest resources inventorying), there is an improvement in the time input efficiencies. Unlike single-purpose inventories, integrated forest resources inventories are more effective due to the less time input spent for the measurement of remaining (marginal) variables.

**Table 7:** Cost-effectiveness of the inventory designs.

C/R	Inventory	n var	Total time input (mts/cluster)	CER (mts/1var)	ICER (mts/1 var)	Inventory pair
BavG	FHI_ICP_	35	300	8.57		
BavG	NFI_2002_	150	840	5.60	4.70	FHI_ICP_–NFI_2002_
SLO	FHI_ICP_	32	344	10.75		
SLO	NFI_1995_	65	494	7.60	4.54	FHI_ICP_–NFI_1995_
SLO	IFRI _2000_	55	418	7.60	3.21	FHI_ICP_–IFRI _2000_
SLO	IFRI _2007_	76	480	6.31	3.09	FHI_ICP_–IFRI _2007_
SWE	FHI_ICP_	45	150	3.33		
SWE	IFRI	250	500	2.00	1.71	FHI_ICP_–IFRI

C/R = Country/Region; Inventory = inventory name; n var = number of variables; total time input = time needed for transportation, walking and measurements in a cluster; CER = cost effectiveness ratio = average time needed for measuring one variable; ICER = incremental cost effectiveness ratio = average time needed for measuring the additional variables; See also abbreviations in Tables 2, 3 and 4.

## Discussion

National forest inventories, integrated national forest resource inventories and environmental monitoring in general are becoming increasingly important tools for decision makers in planning a sustainable future. Information is needed to assess resource availability and environmental impacts; however, the very same information also assists in forestry scenario analysis, whereby the outcome of goods and services at given different management options can be compared, and optimal management schemes can be selected [48].

Whereas conventional forest inventories traditionally have been the responsibilities of individual countries, an increasing number of international agreements highlight the need for international collaboration and for comparable information from all collaborating parties [18]. The already mentioned ICP Forests, operating within the LRTAP convention [14] and occasionally supported by EU legislation [17], was an early example of multi-lateral collaboration. Despite some methodological imperfections [18], controversies associated with air pollution and forest decline [49] and remaining challenges, such as developing indicators for assessing forest biodiversity, as well as the improvement of measurement protocols to be used in monitoring ecosystem processes, ICP Forests has proved to be successful in many areas. Its greatest achievement was perhaps the promotion and support to the participatory countries in building the geographically largest and most complete forest health database in the world [50]. Additionally, with the collected tree defoliation and other data, over the years it has managed to illuminate some of the many effects of tree die-back, some of the relationships between the crown condition and the stress factors [51], as well as the processes related to soils, local climate, local light conditions, etc. Finally, many of the CLRTAP’s International co-operative programs have assisted in bringing to light the CLRTAP’s amended protocols (e.g. Helsinki protocol, Gothenburg protocol [14]), aiming to improve the environment through emission reductions, control of gaseous inorganic and organic compounds, etc.

Perhaps even more collaboration is needed in the implementation of the Conventions on Climate Change [27] and Biodiversity [52]. Because assessing carbon pools and favorable conservation statuses of habitats, habitat types and species, introduced by Habitats Directive [22], requires measuring the sets of variables, much effort will be needed for their harmonization. Consequently, diverse discussions about how to harmonize forest data and how forest inventories should be organized to meet all major user needs [53], [18], [50] are underway.

An additional reason that harmonizing the data is necessary is the use of the same data at different political and spatial levels. As reported [13], a large number of countries already cover the information needs for national policy decisions with specially designed forest inventories and monitoring systems. The very same information is afterwards used by the FAO, which is responsible for preparing regular compilations of the state and development of forests (known as global forest resource assessments) at the global level [26]. At regional levels, processes such as the Montreal Process [54] and Forest Europe [21] compile regional estimates similar to that which the FAO does at the global level.

In addition to improvements due to data-harmonization, our study demonstrates that significant improvements can also be achieved through inventory program integrations. Bavaria, Slovenia and Sweden, for instance, have managed to merge independent national forest and forest health inventories to obtain integrated national forest resource inventories and to end independent, single-purpose inventorying. As described and graphically presented, these integrations were achieved in three different ways:

- by introducing a new NFI grid (in parallel to the old national forest health inventory grid) and the gradual establishment of new sets of subplots for soil-sampling and tree-defoliation assessments in Bavaria (Figure 1);
- by introducing a new set of concentric permanent sample plots (CPSPs) into the existing forest health clusters in Slovenia (Figure 2); and
- by introducing tree-defoliation assessments on the subsets of trees on the cluster’s NFI plots in Sweden (Figure 3).

The results, presented for the core variables, demonstrate that the merging of the inventories has not undermined the precision of the estimates. Moreover, the results are very promising as they allow the detection of rather small differences (e.g. considering the inventory period, 1%–2% change annually) in mean changes and trends in time, which is certainly one of the most important demands imposed on modern inventorying and monitoring [55]–[59]. Furthermore, because all the three integrated forest resource inventories supply data for variables describing growth and yield, site conditions, biodiversity, forest soils, forest health etc., the data are also being successfully used for exploring the relationships and the causalities between the variables [40], [60] and for supporting the international processes along with the FAO reporting. Finally, as shown in Table 7, these integrated inventories also bring financial savings.

However, merging inventories does not necessarily bring only advantages. As Ferretti [50] pointed out and as this study has also demonstrated, the inventory integrations almost always result in cuts of time series. Bavaria, for instance, cut the soil and forest defoliation time series, Slovenia the NFI time series and Sweden cut the forest defoliation time series. Another disadvantage of inventory integration is that the scope of inventories may become too broad and consequently may increase the risk of measurement and assessment errors [61]. This is especially probable if the field crews are not properly trained or they are inexperienced in collecting certain variables.

In the view of the aforementioned ongoing processes, it is also worth considering at what level large-scale inventories should be (if at all) merged. Because forest policy in the EU is the responsibility of individual countries, it seems that the EU nations themselves should develop and run forest assessment systems that meet their specific needs. In concert with this policy, almost all European countries currently perform national forest inventories [13]. However, because the EU also needs forest information for general purposes and for the reporting to the international agreements to which it is a party, this type of information can presently be obtained from the individual countries, provided that the information is properly harmonized.

Naturally, there are also rather different practices around the world. For instance, after long-lasting statewide forest inventorying, the group working at the US Forest Inventory and Analysis Program has managed to merge statewide forest inventories and a nationwide forest health inventory into a common system for the entire country [62], [63]. A similar direction was followed Canada; despite the fact that forest management and policy are to a great extent regulated by the provinces and territories, it managed to create a sampling design across the whole nation [64].

We conclude that presently there is an increasing need for trustworthy information about forests to be used by decision makers within individual countries as well as for international collaboration. This type of information can be cost-efficiently provided if inventories are integrated within individual countries as demonstrated in our study and properly harmonized so that the results can be readily compared between countries and applied to obtain regional and global figures.
